# Current Guideline Risk Stratification and Cardiovascular Outcomes in Chinese Patients Suffered From Atherosclerotic Cardiovascular Disease

**DOI:** 10.3389/fendo.2022.860698

**Published:** 2022-04-28

**Authors:** Sha Li, Hui-Hui Liu, Yuan-Lin Guo, Cheng-Gang Zhu, Na-Qiong Wu, Rui-Xia Xu, Qian Dong, Jie Qian, Ke-Fei Dou, Jian-Jun Li

**Affiliations:** Cardiometabolic Center, State Key Laboratory of Cardiovascular Disease, FuWai Hospital, National Center for Cardiovascular Diseases, Chinese Academy of Medical Sciences, Peking Union Medical College, Beijing, China

**Keywords:** ASCVD, risk stratification, Chinese, high-risk conditions, outcome

## Abstract

**Background and Aims:**

Heterogeneity exists among patients with atherosclerotic cardiovascular disease (ASCVD) with regard to the risk of recurrent events. Current guidelines have definitely refined the disease and we aimed to examine the practicability in Chinese population.

**Methods:**

A cohort of 9944 patients with ASCVD was recruited. Recurrent events occurred during an average of 38.5 months’ follow-up were collected. The respective and combinative roles of major ASCVD (mASCVD) events and high-risk conditions, being defined by 2018 AHA/ACC guideline, in coronary severity and outcome were studied.

**Results:**

The number of high-risk conditions was increased with increasing number of mASCVD events (1.95 ± 1.08 vs. 2.16 ± 1.10 vs. 2.42 ± 1.22). Trends toward the higher to the highest frequency of multi-vessel coronary lesions were found in patients with 1- (71.1%) or ≥2 mASCVD events (82.8%) when compared to those without (67.9%) and in patients with 2- (70.5%) or ≥3 high-risk conditions (77.4%) when compared to those with 0-1 high-risk condition (61.9%). The survival rate was decreased by 6.2% between none- and ≥2 mASCVD events or by 3.5% between 0-1 and ≥3 high-risk conditions. Interestingly, diabetes was independently associated with outcome in patients with 1- [1.54(1.06-2.24)] and ≥2 mASCVD events [1.71(1.03-2.84)]. The positive predictive values were increased among groups with number of mASCVD event increasing (1.10 vs. 1.54 vs. 1.71).

**Conclusion:**

Propitious refinement of ASCVD might be reasonable to improve the survival. Concomitant diabetes was differently associated with the incremental risk among different ASCVD categories, suggesting the need of an appropriate estimate rather than a ‘blanket’ approach in risk stratification.

## Introduction

Atherosclerotic cardiovascular disease (ASCVD) is the leading cause of death worldwide, especially in China (1–3). Traditionally, ASCVD is considered to be a result from the combination of multiple cardiovascular risk factors (RFs). However, the overall risk of ASCVD remains high despite optimal medical management for RFs (4, 5). It is increasingly recognized that heterogeneity exists among patients with ASCVD, which has been defined into the same risk category with a ‘blanket’ approach in the past (6–8). Currently, the paradigm of guidelines has proposed the risk refinement, targeting that the intensity of treatments matches the risk level (9–12). Emerging data strongly indicate that the patients at very-high-risk (VHR) deserves a more veritable approach for clinical management.

Given the disease burden and heterogeneity of ASCVD, we sought to examine the practicability of the newest stratification in a Chinese cohort. Specially, in the present study, we determined 1) the patterns of high-risk conditions, coronary severity, and outcomes among patients with different number of major ASCVD (mASCVD) events at enrolment (0 vs. 1 vs. ≥2 mASCVD events); 2) the associations of coronary severity or outcome with high-risk conditions (0-1 vs. 2 vs. ≥3 high-risk conditions); and 3) the respective and combinative roles of mASCVD events and high-risk conditions, especially diabetes in outcomes.

## Materials and Methods

### Study Design and Populations

Our study complied with the Declaration of Helsinki and was approved by the hospital’s ethical review board (Fu Wai Hospital & National Center for Cardiovascular Diseases, Beijing, China). Informed written consents were obtained from all patients enrolled in this study.

In this observational study with a prospective cohort design, a total of 9944 adults with established ASCVD who were hospitalized in our division of Fu Wai Hospital were consecutively collected from April 2011 through July 2018. Patients with ASCVD were those with coronary artery disease (CAD) including chronic CAD and acute coronary syndrome (ACS), ischemic stroke, and/or peripheral artery disease (PAD) (10). Of the 9944 participants with baseline medical history records, 9806 had CAD, 403 had stroke, and 189 had PAD. Diabetes in this study were all type 2 diabetes. Patients with severe levels of triglycerides (TG, >5.6mmol/L), significant hematologic disorders (white blood cell <3.0 or >10×10^9^), infectious or systematic inflammatory disease, thyroid dysfunction, severe liver/renal insufficiency and/or malignant disease were excluded from the study (**Supplemental Figure 1**).

### Baseline Data Collection and Measurement

Clinical variables of each participant were obtained as described by our previous study (13, 14). The study patients were subjected to elective coronary angiography (CAG). Obstructive CAD defined as the detection of 50-99% diameter stenosis in any of the four major epicardial coronary arteries including left main (LM), left anterior descending (LAD), left circumflex (LCX), and right coronary artery (RCA). Occlusive CAD defined as 100% occlusion of ≥1 coronary artery. The severity and extent of coronary stenosis were assessed using the number of diseased vessels and the Gensini scoring system (15). High-risk conditions and mASCVD events were defined according to 2018 AHA/ACC cholesterol guideline (10).

### Follow-Up and Endpoints Assignment

Follow-up data were obtained at outpatient visits or by telephone contact with every 6-months. We followed-up the cohort mapping for clinical outcomes until the study end date (February 26, 2019, with a window period of 30 days). The primary end points included cardiovascular death, nonfatal myocardial infarction (MI), heart failure (HF), and stroke (13). After all, the data were obtained from 9783 patients and a total of 407 primary events were documented during an average of 38.5 months’ follow-up.

### Statistical Analysis

Statistical analysis was performed with SPSS version 26.0 software (SPSS Inc., Chicago, IL, USA). P values <0.05 were considered statistically significant. Categorical variables were presented as numbers with relative frequencies (percentages) and continuous variables as mean with standard deviation (SDs) or median with inter-quartile range (IQR) as appropriate. Categorical variables were presented as number (percentage) and analyzed by chi-squared statistic test. Differences between groups were determined using the ANOVA or nonparametric test where appropriate. Kaplan-Meier survival curves and Log-rank tests were used to analyze the survival outcomes among the different groups. Cox regression analyses were performed to calculate hazard ratios (HRs) and 95% confidence intervals (CIs) to analyze survival outcome with the high-risk conditions. The covariates including a given high-risk condition and adjusted factors named age, gender, body mass index (BMI), prior percutaneous coronary intervention/coronary artery bypass grafting (PCI/CABG), and current smoking were entered into the multivariable Cox regression model 1, respectively. All covariates above were added simultaneously to the Cox model 2.

## Results

### Baseline Characteristics

**Figure 1:**
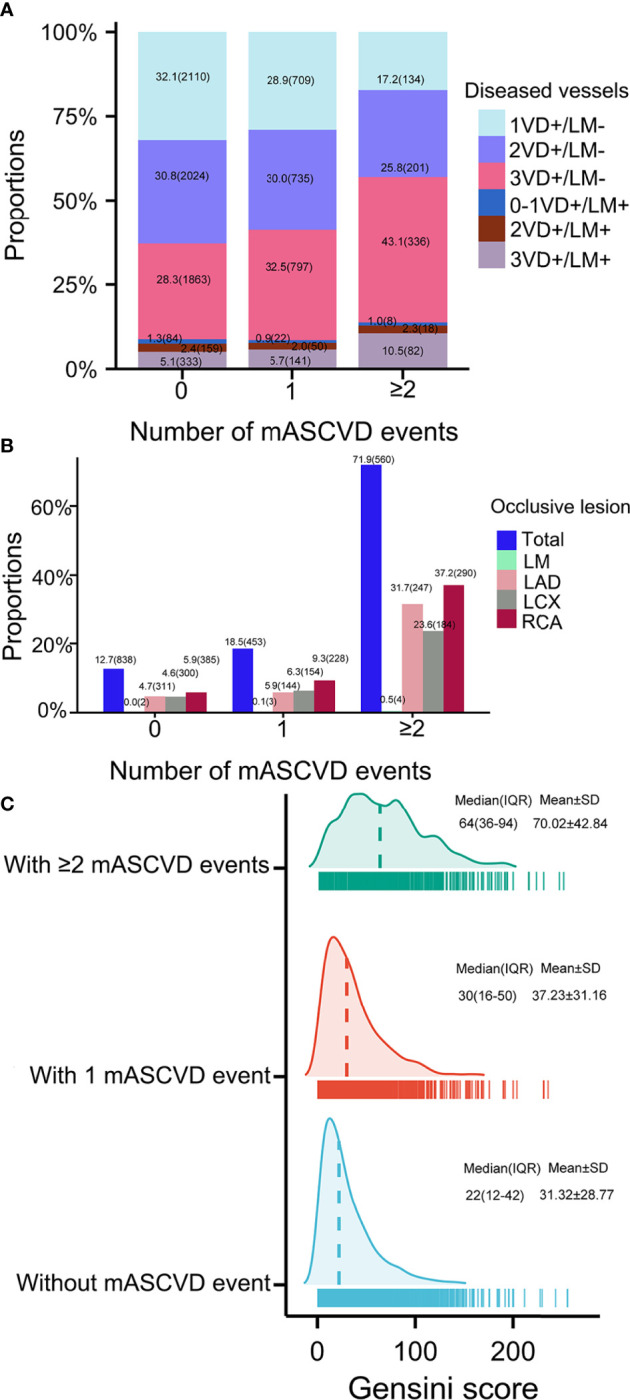
Coronary severity according to number of mASCVD events. Coronary severity was assessed by **(A)** number of diseased arteries, **(B)** occlusive lesion, and **(C)** Gensini score. MASCVD, major atherosclerotic cardiovascular disease; VD, vessel disease; LM, left main; LAD, left anterior descending; LCX, left circumflex; RCA, right coronary artery.

**Figure 2:**
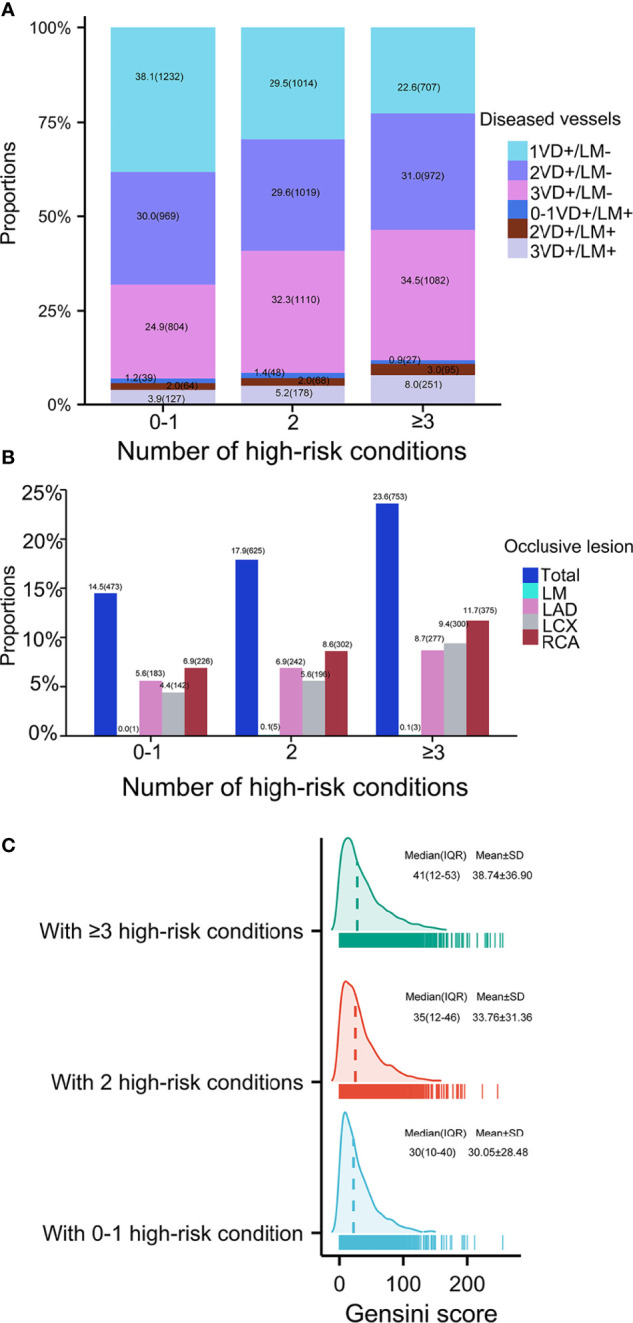
Coronary severity according to number of high-risk conditions. Coronary severity was assessed by **(A)** number of diseased arteries, **(B)** occlusive lesion, and **(C)** Gensini score. VD, vessel disease; LM, left main; LAD, left anterior descending; LCX, left circumflex; RCA, right coronary artery.

Baseline characteristics of patients according to the number of mASCVD events were showed in **Table 1**. Most patients (67.1%) presented with stable event or without mASCVD event followed by patients with 1- (25.0%) or ≥2 mASCVD events (7.8%). Both the number of high-risk conditions (**Supplemental Figure 2**) and the coronary severity (**Figure 1**) were increased with increasing number of mASCVD events. Similarity, we also found that the more high-risk conditions, the severer intensity of coronary lesions (**Figure 2**). The specific descriptions were showed in the **Supplementary Material** section.

**Table 1:** Baseline characteristics by ASCVD categories.

	Without mASCVD event	With 1 mASCVD event	With ≥2 mASCVD events	P for trend
Demographic characteristics				
N	6676	2489	779	
Male,%(n)	67.5(4503)	83.8(2085)	87.3 (680)	<0.001
Age (y)	59.1 ± 8.9	54.7 ± 12.3	56.2 ± 11.7	<0.001
BMI (kg/m2)	25.9 ± 3.2	26.1 ± 3.2	26.0 ± 3.2	0.018
Clinical characteristics				
Hypertension,%(n)	66.6 (4445)	59.9 (1490)	63.4 (494)	<0.001
Hypercholesterolemia*,%(n)	5.5 (367)	6.5 (161)	7.6 (59)	0.026
Diabetes,%(n)	33.2 (2215)	32.2 (801)	36.7 (286)	0.034
CKD3/4,%(n)	3.4 (225)	4.0 (100)	4.9 (38)	0.056
History of congestive HF,%(n)	1.0 (69)	7.2 (179)	18.0 (140)	<0.001
Current smoker,%(n)	33.3 (2220)	46.3 (1152)	46.0 (358)	<0.001
History of PCI/CABG,%(n)	24.1 (1612)	38.2 (951)	41.1 (320)	<0.001
Physical/Laboratory values				
SBP (mmHg)	127.81 ± 16.97	123.91 ± 16.81	123.40 ± 17.53	<0.001
DBP (mmHg)	77.96 ± 10.54	77.20 ± 11.10	76.53 ± 10.84	<0.001
TG (mmol/L)	1.49 (1.09-2.09)	1.52 (1.12-2.13)	1.50 (1.14-2.13)	<0.001
LDL-C (mmol/L)	2.53 ± 0.95	2.44 ± 1.02	2.49 ± 1.01	<0.001
HDL-C (mmol/L)	1.09 ± 0.29	1.01 ± 0.28	0.98 ± 0.26	<0.001
Lipoprotein(a) (mg/L)	142.94 (63.58-351.32)	152.81 (70.07-385.57)	201.82 (80.82-453.40)	<0.001
Glucose (mmol/L)	5.93 ± 1.75	6.00 ± 1.93	6.08 ± 1.98	0.028
HbA1C (%)	6.33 ± 1.10	6.41 ± 1.23	6.52 ± 1.26	<0.001
Cr (umol/L)	77.55 ± 22.35	80.98 ± 18.62	83.31 ± 17.53	<0.001
NT-proBNP (pg/ml)	123.90 (41.70-470.92)	352.75 (81.97-649.67)	494.05 (155.47-883.37)	<0.001
Medications at enrolment				
Anti-platelet,%(n)	61.5 (4109)	69.1 (1720)	69.6 (542)	<0.001
ACEI/ARB,%(n)	16.7 (1117)	23.3 (581)	22.2 (173)	<0.001
β-bloker,%(n)	33.1 (2209)	42.1 (1048)	43.4 338)	<0.001
Statins,%(n)	69.8 (4660)	76.9 (1915)	77.4 (603)	<0.001

Data shown are %(n), mean ± SD, or median (IQR). P values are shown for trend. mASCVD, major atherosclerotic cardiovascular disease; BMI, body mass index; CKD, chronic kidney disease; HF, heart failure; PCI, percutaneous coronary intervention; CABG, coronary artery bypass grafting; SBP, systolic blood pressure; DBP, diastolic blood pressure; TG, triglycerides; LDL-C, low-density lipoprotein cholesterol; HDL-C, high-density lipoprotein cholesterol; HbA1C, hemoglobin A1C; Cr, creatinine; NT-proBNP, N-terminal pro-B-type natriuretic peptide; ACEI, angiotensin converting enzyme inhibitors; ARB, angiotensin receptor blocker.

### Clinical Outcomes

**Figure 3:**
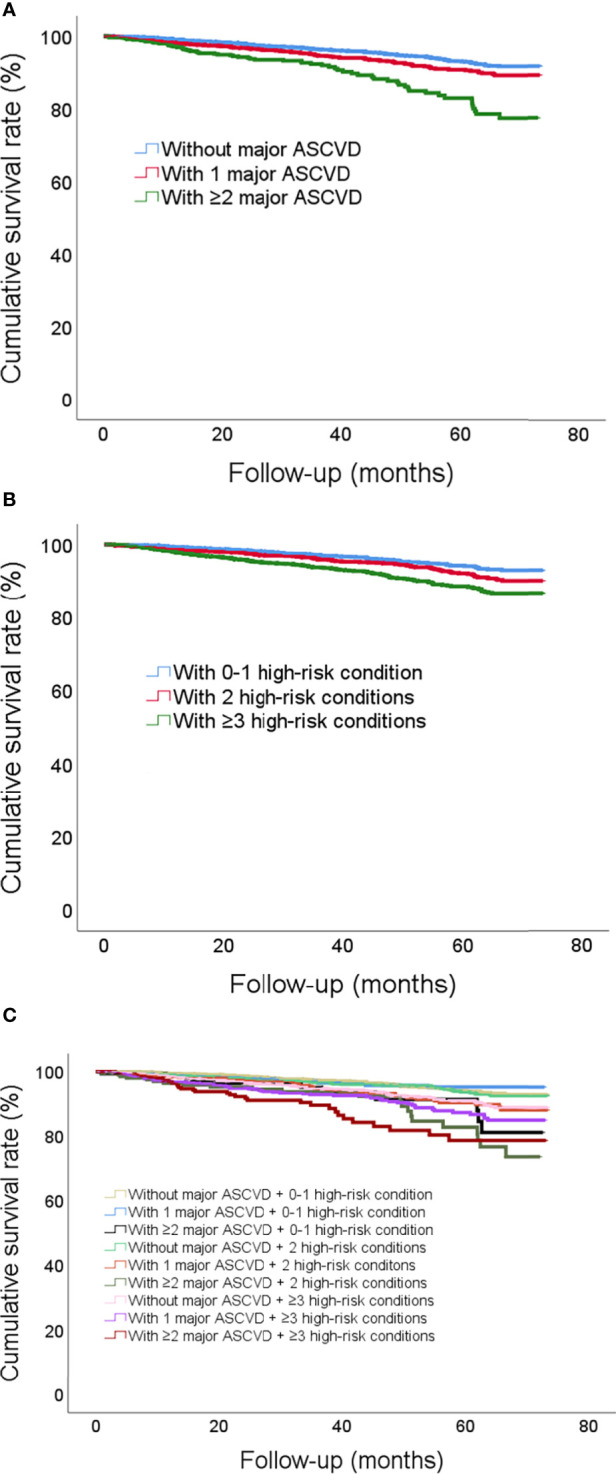
Cumulative incidence curves illustrating the risk of future recurrent events according to **(A)** number of mASCVD events, **(B)** number of high-risk conditions, and **(C)** combinations with number of mASCVD events and high-risk conditions. MASCVD, major atherosclerotic cardiovascular disease.

Patients with multi-mASCVD events were associated with a higher rate of recurrent events (**Figure 3A**). Those with multi-high-risk conditions showed the similar results (**Figure 3B**). As shown in **Figure 3C**, the estimated survival rate was the lowest in patients with ≥2mASCVD events combined ≥3 or 2 high-risk conditions. The highest survival rate was found in patients with none mASCVD events combined 0-1 or 2 high-risk conditions and those with 1 mASCVD events combined 0-1 high-risk conditions. The survival rates in the other combinations ranged from low to high were patients who had 1 mASCVD events and ≥3 high-risk conditions, ≥2 mASCVD events and 0-1 high-risk conditions, 1 mASCVD events and 2 high-risk conditions, and 0 mASCVD events and ≥3 high-risk conditions.

**Figure 4:**
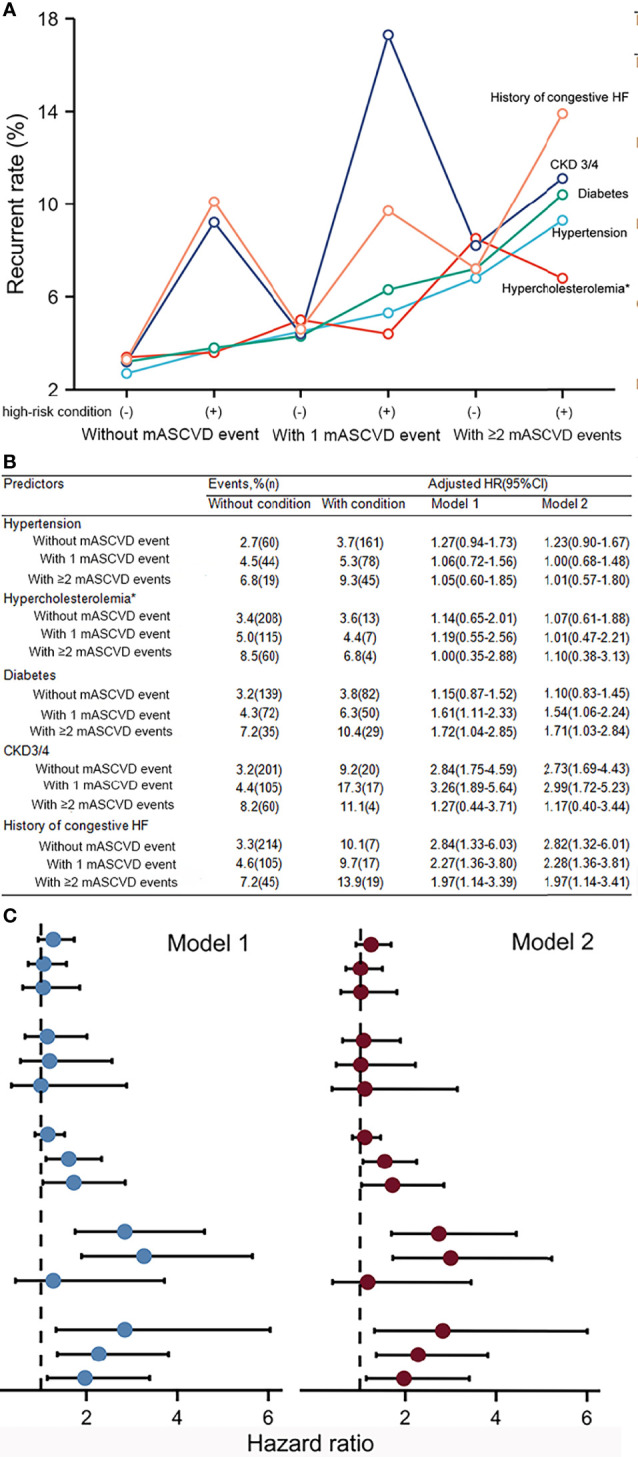
Recurrent event rates **(A, B)** and hazard ratios **(B, C)** for a given high-risk condition among different stages of ASCVD. Model 1 was a given high-risk condition with adjustment for age, gender, BMI, prior PCI/CABG, and current smoking. All covariates including the above covariables and high-risk conditions were added simultaneously to Model 2. MASCVD, major atherosclerotic cardiovascular disease; CKD, chronic kidney disease; HF, heart failure.

The different association of a given high-risk condition with outcome at different ASCVD group was found (**Figure 4**). The rates of recurrent events were stepwise among the exposures to the number of mASCVD events and diabetes (3.2% vs. 3.8% vs. 4.3% vs. 6.3% vs. 7.2% vs. 10.4%). Of note, patients with mASCVD alone (mASCVD+/diabetes-) presented a higher event rate compared to those with diabetes alone (mASCVD-/diabetes+). In the multivariable Cox regression analysis, only diabetes presented a significantly independent risk for outcome in either groups of patients with 1- or ≥2 mASCVD events. The predictive values (HRs) were increased with increasing number of mASCVD events (Model 1, 1.15 vs. 1.61 vs. 1.72, Model 2, 1.10 vs. 1.54 vs. 1.71).

## Discussion

In this study of 9944 patients with documented ASCVD from a Chinese cohort, our findings were substantially important in two respects. First, in baseline analysis, we found that levels of high-risk conditions were far from ideal although the treatment coverage of secondary preventions gradually increased with increasing number of mASCVD events. Moreover, patients with more mASCVD events or high-risk conditions had severer coronary lesions. Clearly, the biology of ASCVD is complex but a majority of the risk can be explained by known modifiable RFs. While the cut-offs of the optimal values are lowering (16–21). Studies have reported a better benefit from successfully recanalized occlusion in patients with multi-vessel CAD compared to those with single-vessel CAD (22, 23). These findings might emphasize the need to focus patients at VHR on RFs achievement and revascularization.

Second, during the follow-up period, we found that the more numbers of mASCVD events or high-risk conditions, the lower the survival rates. The current risk stratification might be appropriate for Chinese and further refinement of ASCVD might be necessary to improve the cardiovascular outcome. Furthermore, we found that diabetes was increasingly associated with risk of recurrent events according to the subgroups of ASCVD. Therefore, individuals with diabetes for risk stratification should be recognized as a patient rather than a ‘blanket’ factor (1).

Current practice guidelines have stratified the subgroup of VHR, a more ominous ASCVD category associated with greater morbidity and mortality (9–12). In fact, identifying those patients at VHR is challenging, as the risk assessment by mASCVD events and high-risk conditions is not adequate. The heterogeneity of mASCVD events for recurrent events might exemplify the necessity of reappraisal (24, 25). Moreover, each high-risk condition possessed different weight for ASCVD risk among different populations (26, 27), underscoring the need for native-data to evaluate the suitable risk stratifications.

The current study had several potential limitations. Firstly, the majority of our patients were CAD, the number of individuals suffered from other ASCVD events was relatively small. Moreover, the definition of high-risk conditions might be not completely accurate. For example, the condition of familial hypercholesterolemia was considered according to clinical diagnosis rather than genetic testing in the present study. Finally, the analysis was a single-center nature and the sampling framework of this study might be not nationally representative.

## Conclusions

The current study might replenish the knowledge of current ASCVD refinement and provide data on Chinese patients. Our results demonstrated that within patients with ASCVD, the number of mASCVD events and/or high-risk conditions was significantly associated with worse patterns of coronary severity and outcome. The weight of a given high-risk condition discriminated across numeracy levels of mASCVD events. Importantly, diabetes was significantly and differently associated with increased risk of the worse outcome among patients at various subgroups of ASCVD.

## Data Availability Statement

The raw data supporting the conclusions of this article will be made available by the authors, without undue reservation.

## Ethics Statement

The studies involving human participants were reviewed and approved by The hospital’s ethical review board (Fu Wai Hospital & National Center for Cardiovascular Diseases, Beijing, China). The patients/participants provided their written informed consent to participate in this study.

## Author Contributions

J-JL have full access to all of the data in the study. SL and J-JL take responsibility for the integrity of the data and the data analysis. Concept and design: J-JL, SL. Acquisition, analysis, or interpretation of data: SL, H-HL. Statistical analysis: SL, R-XX. Patient recruitment: Y-LG, C-GZ, N-QW, QD, JQ, K-FD, J-JL. Drafting the manuscript: SL, J-JL. All authors contributed to the article and approved the submitted version.

## Funding

This work was supported by the Capital Health Development Fund [grant number 201614035] and CAMS Major Collaborative Innovation Project [grant number 2016-I2M-1-011] awarded to Dr. Jian-Jun Li, MD, PhD. The study sponsors did not participate in the study design; the collection, analysis, or interpretation of data; the writing of the report; or the decision to submit the paper for publication.

## Conflict of Interest

The authors declare that the research was conducted in the absence of any commercial or financial relationships that could be construed as a potential conflict of interest.

## Publisher’s Note

All claims expressed in this article are solely those of the authors and do not necessarily represent those of their affiliated organizations, or those of the publisher, the editors and the reviewers. Any product that may be evaluated in this article, or claim that may be made by its manufacturer, is not guaranteed or endorsed by the publisher.
