# Supplementary material for: Current Guideline Risk Stratification and Cardiovascular Outcomes in Chinese Patients Suffered From Atherosclerotic Cardiovascular Disease
Source: Front Endocrinol (Lausanne). 2022 Apr 28;13:860698. doi: 10.3389/fendo.2022.860698 (PMC9096217; doi:10.3389/fendo.2022.860698)

**Supplemental Results**

*Baseline Characteristics*

As showed in Table 1, the oldest age group was those with none- (59.1±8.9 years) and the youngest was those with 1- mASCVD event (54.7±12.3 years). The proportion of male was increased as increasing number of mASCVD events (67.5% vs. 83.8% vs. 87.3%). Mean BMI of patients among 3 groups were all more than 25.0 kg/m^2^.

*High-risk conditions*

In this cohort, hypertension was the leading prevalent co-morbidity (64.7%), the second was current smoking (37.5%) and diabetes (33.2%). Mean number of high-risk conditions was increased with increasing number of mASCVD events (1.95±1.08 vs. 2.16±1.10 vs. 2.42±1.22) but their distributions described by histogram were analogous (supplemental Figure 2). As shown in Table 1, the co-morbidity named diabetes presented a higher proportion among patients with ≥2 mASCVD events than the 2 group of patients with 1- and none- mASCVD event, being not statistically different between the latter 2 groups (36.7% vs. 32.2% vs. 33.2%).

*Severity of coronary disease*

Significant CAD was presented in 6573 (98.5%), 2454 (98.6%), and 779 (100%) patients among respective group of patients with none-, 1-, or ≥2 mASCVD events. There was a significant trend toward higher to highest frequency of multi-vessel disease (Figure 1A), being at least 2VD or LM disease for patients with 1- (71.1%) and ≥2 mASCVD events (82.8%) when compared with those without (67.9%). Furthermore, occlusive lesions (Figure 1B) were detected to be significantly higher to highest in patients with 1- (18.5%) and ≥2 mASCVD events (71.9%) when compared with those without (12.7%). The distributions of Gensini score among each group were showed in Figure 1C, presenting a right shift with an increased medians [22(12-42) vs. 30(16-50) vs. 64(36-94)] and the peaks of the curves from sharp to flat. When patients were divided into 3 groups according to degree of high-risk conditions, significant CAD was presented in 3235 (99.4%), 3437 (98.4%), and 3134 (98.1%) patients among respective group of those with 0-1, 2, or ≥3 high-risk conditions. There was a similar trend toward higher to highest frequency of multi-vessel disease (Figure 2A) for patients with 2 (70.5%) and ≥3 high-risk conditions (77.4%) when compared with those with 0-1 high-risk condition (61.9%). Furthermore, occlusive lesions (Figure 2B) were detected to be significantly higher to highest in patients with 1- (17.9%) and ≥2 mASCVD events (23.6%) when compared with those without (14.5%). The distributions of Gensini score among each group were showed in Figure 2C, presenting a right shift with an increased medians [30(10-40) vs. 35(12-46) vs. 41(12-53)] and a smaller change in the width of the peaks compared to that of Figure 1C.

**Supplemental Figures**

**Supplemental Figure 1** The flowchart of the study. ASCVD, atherosclerotic cardiovascular disease; WBC, white blood cell; ALT, alanine amiotransferase; AST aspartate aminotransferase; eGFR, estimated glomerular filtration rate.


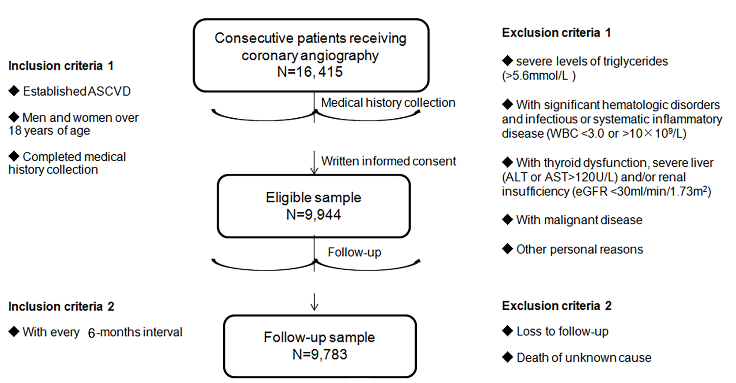


**Supplemental Figure 2** Distribution for number of high-risk conditions according to ASCVD categories.


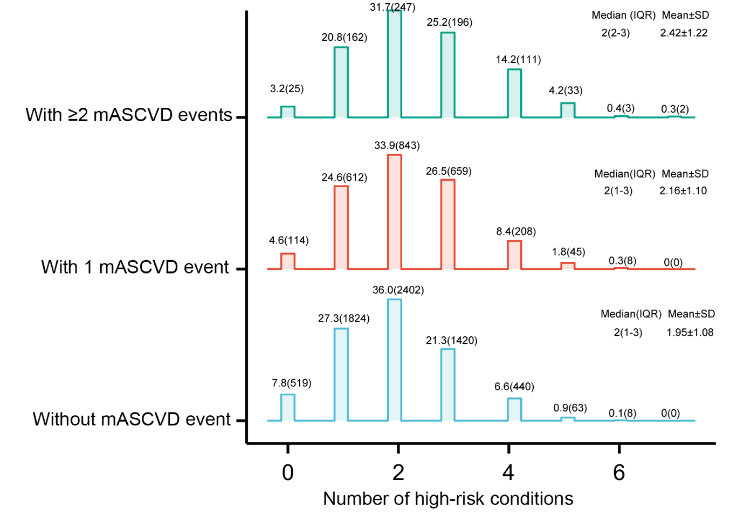

Supplement: Supplementary file 1 [file DataSheet_1.docx]
